# COVID-19 Vaccination Reporting and Adverse Event Analysis in Taiwan

**DOI:** 10.3390/vaccines12060591

**Published:** 2024-05-29

**Authors:** Wan-Chung Hu, Sheng-Kang Chiu, Ying-Fei Yang, Sher Singh

**Affiliations:** 1 Department of Clinical Pathology, Taipei Tzu Chi Hospital, Buddhist Tzu Chi Medical Foundation, New Taipei City 231, Taiwan; wanchung.hu09@tzuchi.com.tw; 2 Department of Medical Research, Taipei Tzu Chi Hospital, Buddhist Tzu Chi Medical Foundation, New Taipei City 231, Taiwan; 3 Department of Biotechnology, Ming Chuan University, Taoyuan 333, Taiwan; 4 Division of Infection Diseases, Department of Medicine, Taipei Tzu Chi Hospital, Buddhist Tzu Chi Medical Foundation, New Taipei City 231, Taiwan; csk33kimo@hotmail.com; 5 School of Medicine, Tzu Chi University, Hualien 970, Taiwan; 6 Division of Infectious Diseases and Tropical Medicine, Department of Medicine, Tri-Service General Hospital, National Defense Medical Center, Taipei 114, Taiwan; 7 Department of Bioenvironmental Systems Engineering, National Taiwan University, Taipei 10617, Taiwan; 8 Department of Life Science, School of Life Science, College of Science, National Taiwan Normal University, Taipei 11677, Taiwan

**Keywords:** SARS-CoV-2, COVID-19 vaccines, vaccine safety, adverse events of special interest (AESIs), vaccine adverse event reporting system (VAERS), vaccine impact

## Abstract

The COVID-19 pandemic necessitated an urgent global response in vaccine deployment, achieving over 70.6% global vaccination coverage with at least one dose. This study focuses on Taiwan’s vaccine administration and adverse event reporting, set against a global backdrop. Using data from Taiwan’s Vaccine Adverse Event Reporting System (VAERS) and global vaccination data, this study investigates vaccine safety and the public health implications of vaccination strategies from local and global perspectives. Taiwan’s proactive approach, resulting in high vaccination rates, provides a case study for the monitoring and management of vaccine-related adverse events. This study offers insights into the safety profiles of various COVID-19 vaccines and further explores the implications of adverse event reporting rates for vaccine policy and public health strategies. The comparative analysis reveals that, while vaccination has been effective in controlling the virus’s spread, safety monitoring remains critical for maintaining public trust. It underscores the necessity of enhanced surveillance and the importance of transparent and tailored risk communication to support informed public health decisions. The findings aim to contribute to the global dialogue on vaccine safety, equitable distribution, evidence-based policy-making, and development of mitigation measures with consideration of local demographics in the ongoing fight against COVID-19.

## 1. Introduction

The COVID-19 pandemic has posed an unprecedented challenge to global public health systems [1,2]. Severe acute respiratory syndrome coronavirus 2 (SARS-CoV-2), the pathogenic coronavirus that emerged in late 2019 from Wuhan in Hubei province in China, is highly transmissible [2]. On infection, the most common symptoms are fever, fatigue, dry cough, and, in severe cases, dyspnea and bilateral lung infiltration [2,3,4]. Therapeutic drugs and human clinical trials have been conducted for symptom treatments [2]. Vaccines were also developed as a proactive control measure. However, as the virus spread rapidly worldwide, the development and deployment of vaccines emerged as cornerstones in combating the pandemic’s reach and severity [5,6,7]. Vaccination has been identified as the most effective measure to control the virus’s proliferation and reduce the morbidity and mortality associated with it [8]. With over 70.6% of the world population having received at least one dose of a COVID-19 vaccine, and 13.53 billion doses administered globally, the impact of these strategies is evident [9]. Additionally, it is important to note the disparities in vaccine distribution, as only 32.9% of people in low-income countries have received at least one dose, reflecting the ongoing challenges in global health equity [9].

However, the expedited rollout of COVID-19 vaccines has also brought vaccine safety into sharp focus. Ensuring the safety of these vaccines is imperative for maintaining public trust and achieving high coverage rates [10,11]. The Vaccine Adverse Event Reporting System (VAERS) has been integral to this effort, providing a mechanism for the timely recording and analysis of any suspected adverse reactions post-vaccination. This active surveillance is pivotal for the early detection of potential safety signals [12,13,14].

Taiwan has been particularly proactive in its vaccination campaign, administering a total of 68,158,988 vaccine doses with high first and second dose rates of 93.8% and 89.06%, respectively, as reported on 26 September 2023 (source: https://covid-19.nchc.org.tw/, accessed on 15 February 2024). As of 30 September 2023, Taiwan had reported 21,225 adverse event notifications, with an average of 31.2 reports per 100,000 vaccine doses administered (source: COVID-19 Vaccine Adverse Event Notification Information Report (1 September 2023–30 September 2023, https://www.fda.gov.tw/, accessed on 15 February 2024)). Specifically, for the Moderna XBB.1.5 vaccine, 24 adverse events were reported out of 861,743 doses, and, for the Novavax vaccine, there were 46 reports out of 642,602 doses, indicating reporting rates of approximately 2.8 and 7.2 events per 100,000 doses, respectively (source: COVID-19 Vaccine Adverse Event Notification Information Report (1 December 2023–31 to December 2023, https://www.fda.gov.tw/, accessed on 15 February 2024)).

This study aims to examine the relationship between vaccine administration and the reporting of adverse events in Taiwan using the data accumulated since the inception of the vaccination program. By analyzing Taiwan’s vaccination strategies and adverse event reports, this research endeavors to provide valuable insights into vaccine safety and contribute to the formulation of evidence-based vaccine policies. Moreover, by juxtaposing Taiwan’s experience with global data, this study seeks to understand the broader implications of different vaccine deployment strategies for the monitoring of vaccine safety.

## 2. Materials and Methods

### 2.1. Data Sources

For global vaccination data, we adopted the publicly available dataset provided by Our World in Data (https://ourworldindata.org/covid-vaccinations, accessed on 15 February 2024). For adverse event reporting after COVID-19 vaccination locally in Taiwan, we utilized data from the reports of COVID-19 adverse events collated from the publicly available Vaccine Adverse Event Reporting System (VAERS). Due to the limitation of the data source for adverse events, the timeframe is from the start of vaccine program to 30 September 2023.

### 2.2. Taiwan COVID-19 Vaccine Program Timeline

**Figure 1:**
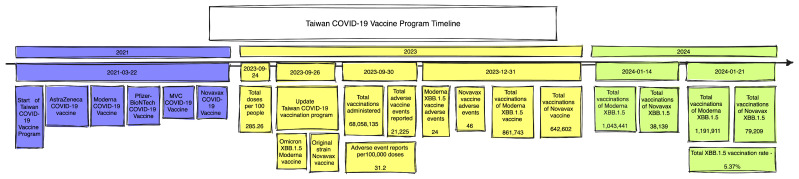
Taiwan COVID-19 vaccine program timeline.

The progression of Taiwan’s COVID-19 vaccination program from its commencement on 22 March 2021 to 21 January 2024 is captured in Figure 1. The timeline shows a structured rollout punctuated by key policy and vaccine development milestones. It details the rollout of various vaccines including AstraZeneca (ChAdOx1 nCoV-19), Moderna (mRNA-1273), Pfizer–BioNTech (BNT162b2 mRNA), MVC (Taiwan MVC-COV1901), and Novavax (NVX-CoV2373), with updates on total doses and vaccination rates. As of 24 September 2023, 285.25 COVID-19 doses had been recorded per 100 people. On 30 September 2023, total vaccinations administered increased to 68,058,135, yet 21,225 adverse vaccine events and 31.2 adverse event reports per 100,000 doses were reported. Significant updates such as the introduction of the Moderna (Spikevax) XBB.1.5 variant (861,743 vaccinations, 24 adverse events recorded) and Novavax (642,602 vaccinations, 46 adverse events recorded) vaccines were also featured for 31 December 2023. Until 21 January 2024, the total number of vaccinations with Moderna XBB 1.5 and Novavax XBB 1.5 were 1,191,911 and 79,209, respectively, with a total XBB 1.5 vaccination rate of 5.37% (Figure 1).

### 2.3. Definition of Adverse Events

Adverse events reported post-COVID-19 vaccination are defined as any health incident reported by individuals following vaccination suspected to be related to or unable to be conclusively dismissed as unrelated to the vaccine administration. The temporal association of these reports with the vaccination does not imply causation. The passive surveillance system aims to statistically assess and analyze factors affecting vaccine safety, such as the manufacturer, batch number, and symptoms of adverse events, to identify potential safety concerns at the earliest opportunity.

Severe adverse events are categorized as per the definitions provided by the International Conference on Harmonisation (ICH) E2A guidelines and the Serious Drug Adverse Reaction Reporting Regulations. Events categorized as severe include outcomes such as death, life-threatening conditions, permanent disability, congenital anomalies in infants, hospitalization or prolonged hospitalization, and other significant clinical events requiring intervention. Definitions of adverse events of special concern are also provided in Supplementary Table S1. Since the TFDA did not provide detailed definitions of adverse events, the information was compiled from Medical Subject Headings (MeSH, https://www.ncbi.nlm.nih.gov/mesh/, accessed on 15 February 2024) or other sources as listed (Supplementary Table S1).

### 2.4. Ethical Approval

The information regarding this study has been made publicly available on the Centers for Disease Control, Ministry of Health and Welfare (https://covid-19.nchc.org.tw, accessed on 15 February 2024) and Taiwan Food and Drug Administration (TFDA, https://www.fda.gov.tw, accessed on 15 February 2024) websites. The data used are anonymized; hence, informed consent from patients is not requisite. This study adheres to the ethical standards set forth in the Declaration of Helsinki (https://www.wma.net/policies-post/wma-declaration-of-helsinki-ethical-principles-for-medical-research-involving-human-subjects/, accessed on 14 May 2023).

## 3. Results

### 3.1. Daily Vaccination Rates

**Figure 2:**
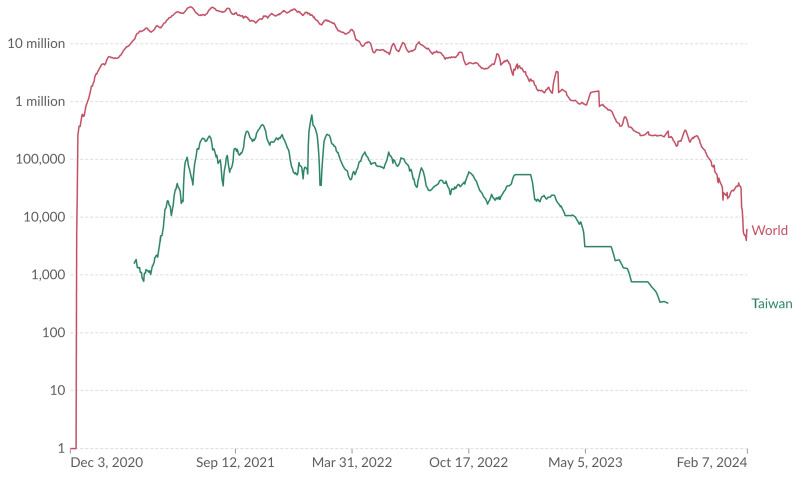
Daily COVID-19 vaccine doses administered with a 7-day rolling average. All doses, including boosters, are counted individually. (Results were analyzed by Our World in Data (https://ourworldindata.org/explorers/coronavirus-data-explorer?yScale=log&zoomToSelection=true&facet=none&uniformYAxis=0&country=OWID_WRL~TWN&pickerSort=asc&pickerMetric=location&hideControls=false&Metric=Vaccine+doses&Interval=7-day+rolling+average&Relative+to+Population=false&Color+by+test+positivity=false, accessed on 14 February 2024) based on the original study [15]).

The trend in daily COVID-19 vaccine doses administered in Taiwan, as compared to global efforts, is revealed in Figure 2. This comparison shows distinct peaks both in local and global administration rates, aligning with policy changes and vaccine availability.

Figure 2 presents global and Taiwan trends in daily COVID-19 vaccine doses administered, with a 7-day rolling average of doses administered, including all vaccine doses and boosters. The red curve represents the global trend, while the green curve pertains to Taiwan. Data points, such as 11.69 million doses globally and 1578 doses in Taiwan on 22 March 2021, and approximately 271,614 doses globally and 323 doses in Taiwan on 24 September 2023, illustrate that the trends in the world and Taiwan were consistent at the COVID-19 outbreak, at the start of the vaccine program, at the second and third dose, and at the following boosters.

### 3.2. Vaccination Coverage

**Figure 3:**
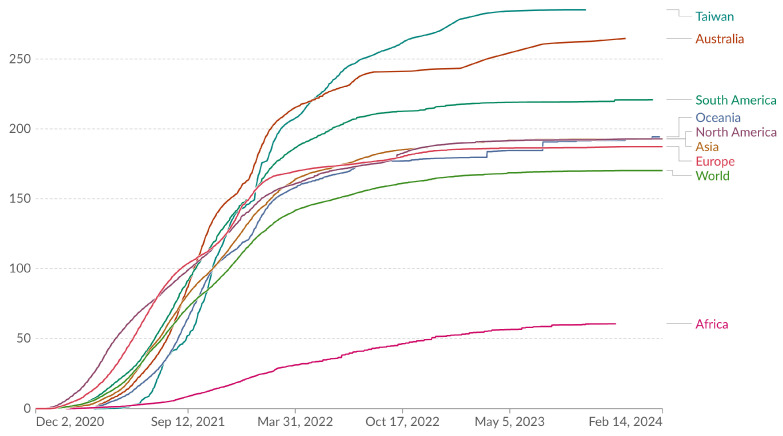
Total COVID-19 vaccine doses administered per 100 people. All doses, including boosters, are counted individually. (Results were analyzed by Our World in Data, “Cumulative COVID-19 vaccinations per 100 people” [dataset] (https://ourworldindata.org/grapher/covid-vaccination-doses-per-capita?country=TWN~OWID_ASI~OWID_WRL~OWID_EUR~OWID_OCE~OWID_NAM~AUS~OWID_AFR~OWID_SAM, accessed on 15 February 2024) based on the original study [15]).

**Figure 4:**
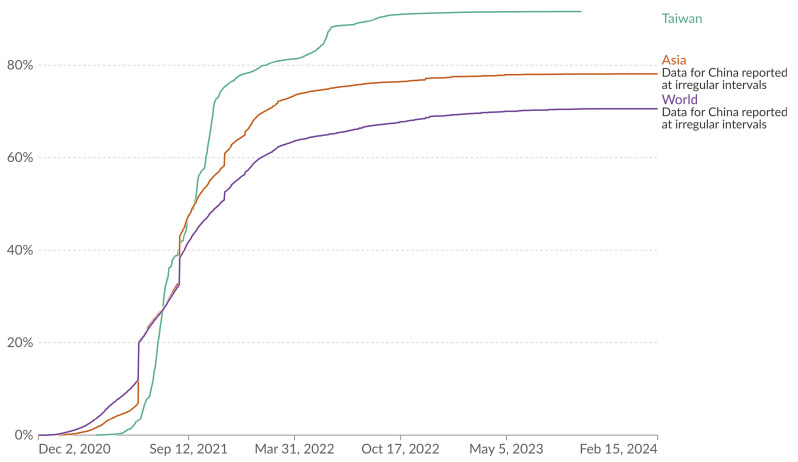
Share of people who received at least one dose of COVID-19 vaccine. Total number of people who received at least one vaccine dose, divided by the total population of the country. (Results were analyzed by Our World in Data, “people_vaccinated_per_hundred” [dataset] (https://ourworldindata.org/grapher/share-people-vaccinated-covid?country=TWN~OWID_WRL~OWID_ASI, accessed on 16 February 2024) based on the original study [15]).

**Figure 5:**
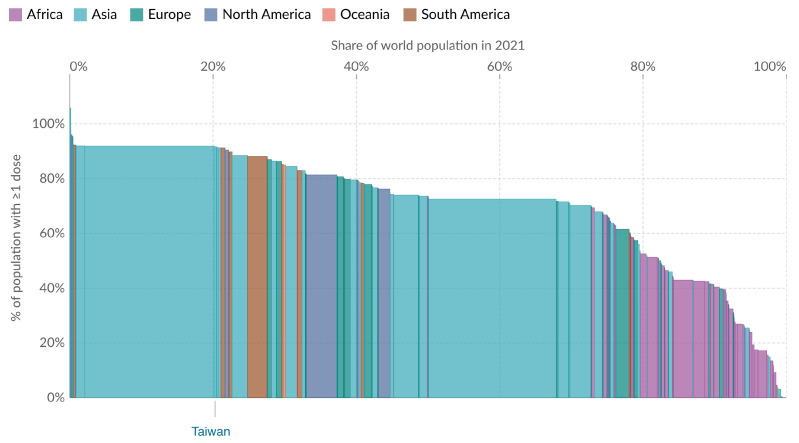
COVID-19 vaccination coverage worldwide. Share of people who received at least one dose of COVID-19 vaccine. (Results were analyzed by Our World in Data, “% of population with ≥1 dose” [dataset] (https://ourworldindata.org/grapher/covid-people-vaccinated-marimekko), accessed on 16 February 2024).

Assessment of the total doses administered and the proportion of the Taiwanese population with at least one vaccine dose are presented in Figure 3 and Figure 4. In addition, Figure 5 offers a global perspective, illustrating Taiwan’s vaccination coverage in relation to worldwide data as of 22 January 2024.

Pertaining to global and regional trends in COVID-19 vaccine doses administered per 100 people from 2 December 2020 to 23 January 2024, this graph demonstrates the per capita vaccine doses administered since Taiwan initiated its COVID-19 vaccination program on 22 March 2021, including booster doses. Each curve represents the vaccination trajectory for different regions. Initially, North America administered 25.92 doses per 100 people, while Taiwan started at 0.01 doses. By 17 September 2023, Taiwan had markedly escalated its rate to 285.25 doses per 100 people (Figure 3).

Figure 4 displays the share of the population in Taiwan, Asia, and globally that received at least one dose of the COVID-19 vaccine, covering the period from 2 December 2020 to 23 January 2024. The vaccination proportion for Taiwan reached 91.7% by 17 September 2023. The overall vaccination shares for Asia (including data for China reported at irregular intervals) and the world are 78.1% and 70.5%, respectively, as of the same date. This chart shows the share of the total population that has received at least one dose of the COVID-19 vaccine, which may not be equal to the share with a complete initial protocol if the vaccine requires two doses. If a person receives the first dose of a two-dose vaccine, this metric goes up by 1. If they receive the second dose, the metric stays the same. Overall, by May 2023, Taiwan had the highest share of people receiving at least one dose of vaccine.

Figure 5 illustrates global COVID-19 vaccination coverage in different geographic regions of the world. This bar chart shows the percentage of the population that has received at least one dose of the COVID-19 vaccine, with different colors in the chart indicating various regions. Taiwan accounts for 0.3% of the world’s population. The data point shows that at least 91.65% of Taiwan’s population received one dose by 24 September 2023, indicating that its vaccination rate is ahead of many countries and regions.

### 3.3. Public Health Outcomes and Concerns

**Figure 6:**
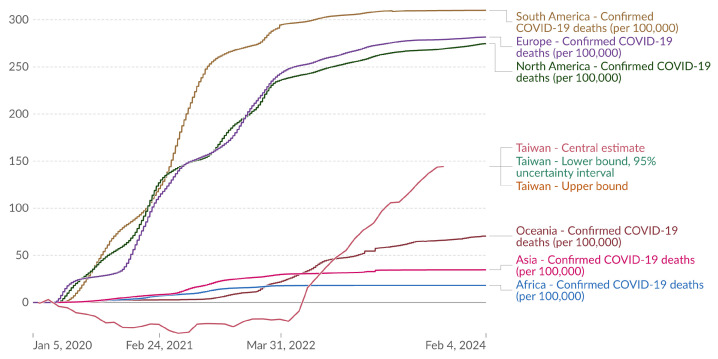
Estimated cumulative excess deaths per 100,000 people during COVID-19, Taiwan. (Results were analyzed by Our World in Data (https://ourworldindata.org/explorers/coronavirus-data-explorer?tab=chart&zoomToSelection=true&facet=none&coutry=TWN~OWID_EUR~OWID_ASI~OWID_OCE~OWID_SAM~OWID_AFR~OWID_NAM&pickerSort=asc&pickerMetric=location&hideControls=true&Metric=Excess+mortality+%28estimates%29&Interval=Cumulative&Relative+to+Population=true&Color+by+test+positivity=true) based on The Economist (2022) and the WHO COVID-19 Dashboard, accessed on 16 February 2024). For countries that did not report all-cause mortality data for a given week, an estimate is shown, with uncertainty interval. If reported data are available, that value only is shown. On the map, only the central estimate is shown.

The estimated cumulative excess deaths per 100,000 people in Taiwan during the pandemic are evaluated and depicted in Figure 6. Complementing this evaluation, Table 1 presents an analysis of the basic information of adverse event notifications reported between 22 March 2021 and 30 September 2023, providing a foundational understanding of the adverse events recorded. The risk of serious adverse events is assessed across different age groups, showing a higher rate of notifications, particularly death cases, in individuals aged 65 and above (Table 1). Further, the impact of vaccination on different age groups is elucidated in Table 2, which categorizes death notifications by age group, offering a granular view of mortality in the context of the vaccination campaign.

Excess deaths refer to the discrepancy between the actual and expected number of deaths during a specific period. The pink curve represents the central estimate for Taiwan, with a notable data point indicating an estimated 144.28 deaths per 100,000 people on 18 September 2023, which is not comparable to the reported death cases (1628) from TFDA since Our World in Data provides overall cumulative excess death cases from either COVID-19 infection pre- or post-vaccinations or co-occurrence of vaccination and infection, whereas TFDA provides COVID-19-vaccination-specific death events (Figure 6; Table 1). After 26 September 2022, the estimated excess deaths in Taiwan exceeded those of Oceania, Asia, and Africa. The slope of cumulative excess death of Taiwan increased suddenly after 31 March 2022, contrasting to those of South America, Europe, and North America, which had more flat curves in the same time period (31 March 2022 to 4 February 2024), highlighting the diverse impacts of the pandemic across different regions. The 95% uncertainty intervals are omitted as the central estimate aligns with the upper and lower bounds. Data are sourced from *The Economist* (2022) and the WHO COVID-19 Dashboard.

### 3.4. Adverse Events Analysis

**Figure 7:**
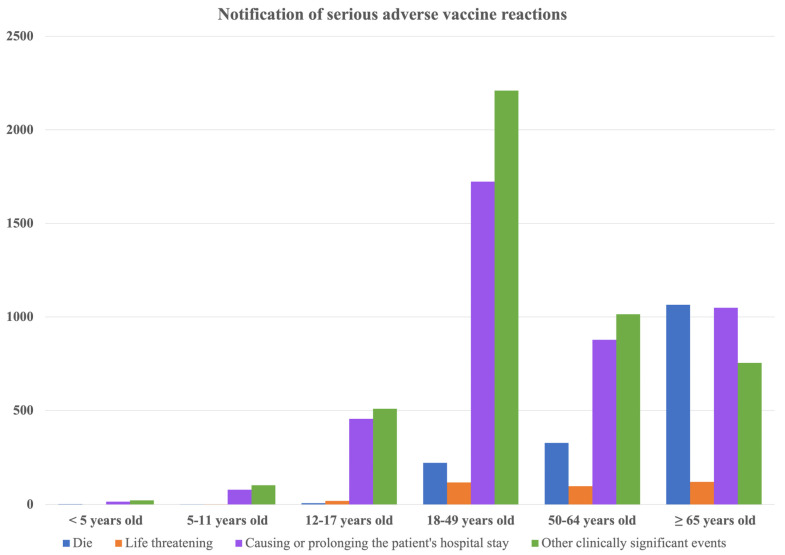
Notification of serious adverse vaccine reactions in Taiwan from 22 March 2021 to 30 September 2023. Data source: COVID-19 Vaccine Adverse Event Notification Information Report, Taiwan Food and Drug Administration, https://www.fda.gov.tw/tc/includes/GetFile.ashx?id=f638331478640715627&type=2&cid=45553, accessed on 15 February 2024.

Table 3 and Table 4 first provide a detailed account of the cumulative trends of adverse events of special interest, including the data related to the Moderna Omicron XBB.1.5 and Novavax vaccines, offering a foundational overview of these occurrences throughout the study period. Following this, Figure 7 illustrates the serious adverse reactions to vaccines in Taiwan, covering the period from 22 March 2021 to 30 September 2023. This bar chart illustrates the number of serious adverse reactions to COVID-19 vaccination reported across various age groups, including death (dark blue), life-threatening conditions (orange), causing or prolonging hospitalization (purple), and other clinically significant events (green). The data indicate that the highest number of hospitalization-related reports occurred in the 18–49 age group, while reports of death were significantly higher in the group aged 65 and above. Reported cases of serious reactions for specific types of vaccine are also listed in Table 5, Table 6 and Table 7.

**Figure 8:**
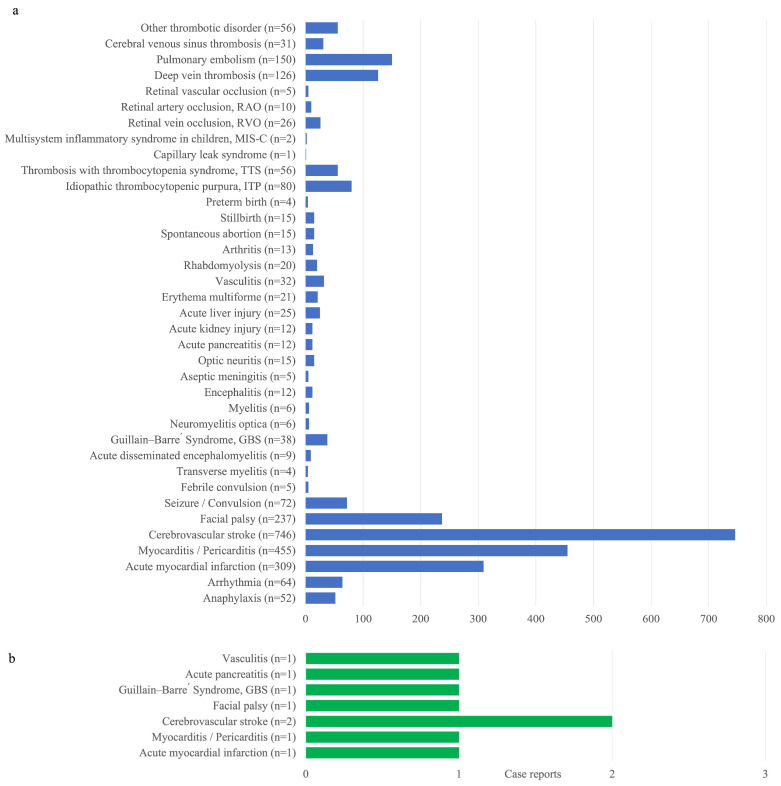
Cumulative reporting of adverse events of special concern in Taiwan in the period of (**a**) 22 March 2021 to 30 September 2023 and (**b**) 12 January 2023 to 31 December 2023. Data source: COVID-19 Vaccine Adverse Event Notification Information Report, Taiwan Food and Drug Administration.

Figure 8 enhances the detailed tables by graphically highlighting the trends of adverse events of special interest (AESIs) with definitions provided in Supplementary Table S1. Figure 8a encompasses reports from 22 March 2021 to 30 September 2023, including reports on thrombotic disorders such as renal infarction, ischemic bowel diseases, portal vein thrombosis and iliac vein, intestinal vascular venous thrombosis, and superior mesenteric artery thrombosis. Notably, cerebrovascular stroke (746 cases), myocarditis/pericarditis (455 cases), and acute myocardial infarction (309 cases) were the most frequently reported conditions, highlighting the need for particular attention to these conditions following vaccination. Other significant reported adverse events include anaphylaxis (52 cases) and arrhythmia (64 cases). Figure 8b includes reports from 26 September 2023 to 31 December 2023 for the monovalent Moderna XBB.1.5 vaccine containing the Omicron XBB.1.5 variant antigen and the monovalent Novavax vaccine containing the original strain antigen.

Reported symptoms include left renal infarction, ischemic intestinal disease, hepatic portal vein thrombosis and intestinal bone veins, intestinal vascular venous thrombosis, upper mesenteric artery embolism, renal vein thrombosis, splenic infarction, acute venous thromboembolism of unspecified upper limbs, lower limbs arterial occlusion, distal upper limb artery thrombosis, renal artery infarction, and carotid artery embolism (22 March 2021~30 September 2023).

### 3.5. Comparative Analysis of Adverse Events across Vaccine Types

The adverse event rates post-vaccination among different manufacturers are comparatively analyzed in Supplementary Tables S2–S4. These tables indicate that the AstraZeneca vaccine reported the highest number of serious adverse events post-basic dose, followed by the Moderna and BioNTech vaccines. The rates of serious adverse events following booster doses were also higher for the Moderna vaccine. This information is pivotal in understanding the safety profiles of different vaccines and tailoring vaccination strategies.

As of 8 February 2023, a total of 66,676,951 doses of vaccines had been administered, including 15,297,919 doses of AstraZeneca, 23,997,476 doses of Moderna, 3,069,103 doses of Taiwan MVC vaccinations, 19,708,850 doses of BioNTech vaccinate, and 556,156 doses of Novavax. Among all the reported adverse events, 1612 cases in total (656 women, 956 male) were observed from 1 February 2023 to 8 February 2023. Without considering the causality from vaccination, AstraZeneca had a relatively higher number of suspected serious adverse events, adverse events, and non-serious adverse events when compared to other manufacturers during this period (Table 8).

## 4. Discussion

### 4.1. Safety Profile across Vaccines

Large differences in observed rates of adverse events of special interest (AESIs) across age groups and genders indicate the need for stratification or standardization before using background rates for safety monitoring. There is considerable population-level heterogeneity in AESI rates between databases [16]. A prospective observational study from northern India has indicated that AESIs associated with the ChAdOx1-nCoV-19 vaccine are more common among females, individuals with hypothyroidism, diabetics, or those who had a history of COVID-19 prior to vaccination. Females and individuals with hypothyroidism also face a higher risk of persistent adverse events. Particular attention needs to be paid to AESIs such as arthropathy, recurrent viral infections, and severe dengue fever. Future studies should explore the role of gender-related and hormonal differences in the occurrence of COVID-19-vaccine-related adverse events. Considering that a significant proportion of COVID-19 infections and non-trivial rates of AESIs occur post-vaccination, there needs to be a strategic reconsideration of blanket recommendations for mass vaccination. Women, individuals with comorbidities such as hypothyroidism, and those with a history of COVID-19 should be informed about the protective benefits of vaccines as well as the risks of adverse events post-vaccination. Compared to further large-scale promotion, an individualized vaccination strategy may be a better choice for public health safety [17].

In addition, clinical trials may not observe participants for enough time to detect all possible side effects. Knowledge about vaccine-related adverse reactions is incomplete, and risk assessment may vary from person to person, particularly for those with genetic immune issues that could increase risk. Such instances are uncommon and typically found only in post-market analysis [18,19]. Also, the lack of definitive proof linking COVID-19 vaccinations to the majority of cases is a critical issue necessitating further research to determine any causative connections [20]. In Victoria, Australia, a clinical surveillance study on the long-term follow-up and outcomes of COVID-19-vaccine-associated myocarditis revealed that symptoms persist for many patients for up to 180 days after the onset of the AESI. Notably, male patients frequently exhibit quicker symptom resolution, even with initially higher troponin levels, underscoring a gender-specific disparity in recovery. This observation underscores the critical need for further research and prolonged follow-up to understand the implications fully [21]. In Taiwan, it has been observed that certain AESIs can manifest over an extended period, sometimes spanning several hundred days, highlighting the necessity for ongoing monitoring.

Following the Emergency Use Authorization (EUA), significant overlap between injuries from mRNA vaccine products and both Post-Acute Sequelae of SARS-CoV-2 Infection (PACS) and severe acute COVID-19 illnesses has been observed, often concealing the vaccine’s role in causing these conditions. Moreover, frequent mRNA booster injections may paradoxically impair immune function, increasing the likelihood of COVID-19 infection. Alongside this, a number of serious AESIs like deaths, cancers, cardiac issues, and various other health disorders have been reported, raising substantial concerns. These concerns are particularly acute for the elderly and point to an urgent need to investigate the link between vaccines and AESIs, as well as the issues related to DNA contamination and abnormal protein production associated with the mRNA vaccines [22].

Moreover, when considering regional differences, the incidence rates of adverse events of special interest such as deep vein thrombosis and pulmonary embolism in Scotland were notably higher during the pandemic period [23]. These regional data underscore the variability in vaccine response and the importance of contextual analysis. On the other hand, the ChAdOx1 vaccine has been associated with a higher number of adverse reaction events, specifically drawing attention to an immune-mediated mechanism termed vaccine-induced thrombocytopenic thrombosis (VITT) [24,25]. This condition has manifested with a particularly higher risk profile for cerebral venous thrombosis (CVT) in women following ChAdOx1 vaccination [24], which emphasizes the need for gender-specific risk assessment and communication.

Given these diverse findings, the imperative to communicate risks and corresponding mitigation measures with greater detail, clarity, and care becomes evident. This approach is essential for ensuring the public understands the current risks associated with vaccination and can make informed decisions regarding their health.

### 4.2. Risk Management in Vaccination

In patients with Post-Acute Sequelae of SARS-CoV-2 Infection (PACS), the presence of SARS-CoV-2 proteins and mRNA has been detected in tissue samples. Studies have reported the detection of the SARS-CoV-2 nucleocapsid and spike proteins in the appendix of an individual with PACS and lymphoid hyperplasia of the appendix as long as 426 days after the onset of symptoms. Furthermore, the nucleocapsid protein of SARS-CoV-2 was also found in skin tissue [26,27]. These findings emphasize the critical need for advanced surveillance of adverse reactions, especially those that may have a delayed onset. The possibility that reactions could manifest several hundred days after vaccination necessitates vigilant and long-term monitoring. Effective risk communication is essential in fostering public confidence in vaccine safety. Openly sharing risk management data and measures for post-vaccination adverse events enables the public to make informed health decisions [28].

In Singapore, increased rates of myocarditis/pericarditis, appendicitis, and CVT linked to COVID-19 mRNA vaccines have been observed [29]. It is imperative to educate patients on the expected reactions to a homologous COVID-19 mRNA vaccine booster, which are generally less frequent and severe than those following the second vaccine dose [30]. An individualized approach to vaccination may enhance public health safety, particularly for specific demographics, including women, individuals with certain comorbidities, and those with previous COVID-19 infections [17].

### 4.3. Policy Implications

The adverse event information provided by the TFDA can only reflect the ‘reported’ cases of death but cannot be conclusive on the reasons for the higher excess deaths observed after 31 March 2022. Whether the mutation of the virus and/or the policy implementation is causative should be further explored. To mitigate the risk from the perspective of the vaccine program, development of evidence-based recommendations is key to enhancing the safety and efficacy of vaccines. Such recommendations could extend to improving surveillance systems to better capture adverse events, which is crucial for the timely detection and response to potential vaccine-related issues [28]. Enhanced surveillance not only aids in immediate risk management, but also contributes to the long-term goal of strengthening vaccine safety systems [31]. Transparency in reporting and the tailoring of public health communications are also fundamental to maintaining and building vaccine confidence [28].

It should be noted that providing the public with a clear understanding of vaccination risks and the measures in place to mitigate them is essential [32]. This understanding becomes the foundation upon which individuals can make informed decisions regarding their health and vaccination choices. Furthermore, it is critical to recognize and address demographic-specific responses to vaccines. The young and elderly populations may react differently to vaccines, necessitating adjustments in vaccination strategies and post-vaccination monitoring for these groups [33]. Policy frameworks could be refined to ensure rigorous post-vaccination monitoring, especially in age groups or populations that have reported higher incidences of adverse reactions [33].

In light of the dynamic landscape of COVID-19 and the occurrence of breakthrough infections, there is a call for strategic reconsideration of blanket mass vaccination policies. An in-depth analysis of the rates of adverse events of special interest within the vaccinated population should inform such policy revisions, ensuring that recommendations are nuanced and tailored to the evolving epidemiological context [17]. The integration of these insights into policy-making will ensure that vaccination programs are not only reactive to current trends but also proactively designed for resilience and public trust in the face of future challenges.

This study highlights the importance of enhanced surveillance systems capable of detecting and analyzing subtle changes in adverse events promptly. Regular updates on vaccine efficacy and emerging variants are recommended. Surveillance systems must be dynamic and responsive, capable of adapting monitoring strategies to the evolving nature of vaccines and pathogens. Particular attention should be paid to highlighted conditions like myocarditis/pericarditis and stroke, with a focus on vulnerable demographics to tailor public health responses [24].

### 4.4. Further Research Needs

The identification of research gaps highlights a significant need for future investigations into the long-term safety and efficacy of different COVID-19 vaccine brands and types [34]. There is a particular necessity to understand the vaccine response among diverse ethnic groups, which is crucial for tailoring vaccination strategies appropriately [35,36]. Future studies should prioritize assessing the durability of immunity conferred by vaccines over extended periods [37,38]. This includes investigating how long immunity lasts following the full initial vaccination series and subsequent booster doses. Moreover, it is essential to understand the robustness of vaccine-induced protection against the continuously evolving virus variants, which may differ in virulence and transmissibility [39,40,41].

Additionally, research should not only encompass broad population-wide analyses but also focus on specific populations that may present unique responses or higher risks of adverse events [42]. This could involve individuals with underlying health conditions, different age brackets, or varying exposure risks. Precision medicine research could be a strategy to disclose the individual vaccine-related adverse effects via tools like single-nucleotide polymorphism. The polymorphism of major histocompatibility complex (MHC) should be analyzed to find out the association between specific MHC haplotypes and vaccine-related adverse effects. Through this approach, immune pathogenesis after vaccine inoculation can be explored, and then we can predict possible specific adverse effects for certain individuals before vaccine injection. Thus, we can choose which kind of vaccine, like DNA vaccine, RNA vaccine, or protein vaccine, is most suitable for each individual. Therefore, possible severe adverse effects after vaccination can be avoided.

By addressing these research needs, future studies can contribute to the development of vaccines that are both effective and safe for all segments of the population [43]. Such work will be instrumental in guiding public health decisions and maintaining confidence in vaccination programs as an essential component of global health initiatives.

### 4.5. Significance and Limitations

We wanted to tell a story by comparing vaccination rates locally in Taiwan with those in other world regions to give the overall conclusion that Taiwan has a relatively higher vaccination rate (at least one vaccine dose) compared with Asia or the rest of the world. Based on this context, we provided reported cases stratified by different age groups. Among all age groups, we found that the 18–50 years age group had the highest number of reported cases under the same classification of adverse events compared to other age groups. However, a higher number of death notifications could be observed in groups with higher age ranges. Adverse events with the highest number of reported cases were found to be, in the following order, cerebrovascular stroke, myocarditis/pericarditis, and acute myocardial infarction, of which one is in accordance with previous studies that stated that myocarditis is the most reported adverse event [44,45]. Therefore, the higher vaccination coverage in Taiwan was presented as a context to link with the reported adverse events in Taiwan to further explore whether there are any underlying factors that may affect the different profiles of adverse events in local regions, which could include demographic composition or policy implementations.

One of the critical points we wanted to address is the exploration of the underlying reasons that could be potentially related to reported adverse events from vaccination from a local perspective. Amer et al. [46] supports our point that more attention should be directed toward countries with political conditions that hinder the vaccination process while cautiously interpreting current vaccination results. In addition, at this point, the COVID-19 pandemic is not a major issue and concern because of better implementations of control measures in the past few years. However, it is at this point that we need to raise much more concern for the side effects or adverse events based on the VAERS. Seneff and Nigh [47] suggested that re-evaluation of COVID-19 vaccines is required based on the fact that it is an unprecedented vaccine; a vaccine normally takes 12.5 years to develop, yet it took a relatively short period of time to develop COVID-19 vaccines with reported high efficacy (90–95%).

On the other hand, we also compared the reported adverse events between vaccinations for COVID-19 and influenza. For the COVID-19 vaccine, 31.2 adverse events were reported per 100,000 doses administered. For the influenza vaccine, 3.99 adverse events were observed per 100,000 doses administered [48]. It was also mentioned that the influenza vaccine (RIV4) generally is safe among the Taiwan population when compared to the overall safety profiles for the influenza vaccine in this population [48]. Since the adverse event datasets were both adopted from the VAERS and both have limitations in determining the causality from the vaccines, it would be comparable and reasonable to state that the COVID-19 vaccine has potentially higher reported adverse events than other types of vaccine.

In Taiwan, there has been a significant variation in the cumulative observation time for different brands of COVID-19 vaccines, and there are also substantial differences in the basic characteristics (such as age) of the populations vaccinated with each brand. Data on adverse events related to COVID-19 vaccines are solely derived from the reports received by the Vaccine Adverse Event Reporting System (VAERS) of the Centers for Disease Control. One of the limitations of the VAERS is that it recorded reported adverse events without elucidating the number of doses or different brands of vaccine under the stratifications of various adverse events of special concern, age ranges, or time of onset. An adverse event report refers to any incident reported voluntarily by the notifier at any time post-COVID-19 vaccination due to suspicion of or an inability to rule out a connection with the vaccine administration. Although these reported incidents occur after the administration of the vaccine, they do not imply causation by the vaccine itself. In addition, the adverse event data are not categorized by employing the GRADE system. The current VAERS also lacks a control vaccine (e.g., influenza vaccine), which would have been required to identify the higher risks of SARS-CoV-2 vaccines in this study. All of these limitations implicate the potential impacts of policy implementations with different timeframes of vaccinations and different vaccine brands, leading to the complexity of elucidating the causations of adverse events including different brands of vaccine, doses, demographics, or other contributing factors.

Taken together, despite the limitations in investigations of causation between the vaccine and reported adverse events, and the lack of GRADE system and control vaccine in the VAERS, the purpose of the passive safety surveillance conducted through the spontaneous adverse event reporting system is to statistically assess and analyze factors affecting vaccine safety, such as the demographics, vaccine manufacturer, batch number, and symptoms of adverse events, to identify potential safety concerns at the earliest opportunity. We thus provide an insight from global to local perspectives on how to best utilize local adverse event reporting systems to address the importance of paying more attention to characterizing the causation between these adverse events and vaccination and implementing better surveillance systems and refinement of policies by tailoring personal requests for different types of vaccines.

## 5. Conclusions

Our study’s systematic examination within Taiwan’s context—set against the backdrop of worldwide COVID-19 vaccination campaigns—has brought to light the critical need for vigilant safety monitoring. The observed disparity in adverse events among different vaccines accentuates the variability in vaccine safety profiles and reinforces the value of robust reporting systems like VAERS. These systems play a crucial role in the early detection of safety signals, thereby ensuring the safe continuation of vaccination programs. The collected data provide pivotal insights into risk management and the development of vaccine policies that are specifically tailored to demographics exhibiting higher rates of adverse events. As we move forward, the enhancement of surveillance systems, the refinement of policy frameworks for improved post-vaccination monitoring, and the commitment to transparent communication will be fundamental in maintaining public trust in vaccination efforts and overcoming the challenges of global health equity.

## Figures and Tables

**Table 1:** Analysis of basic information of adverse events with notification reported cases from 22 March 2021 to 30 September 2023.

Classification of Reported Cases		Number of Reported Cases						
	Age Group (Years)	<5	5–<12	12–<18	18–<50	50–<65	≥65	Sum
	Death	2	1	7	222	328	1065	1628
	Life-threatening condition	0	1	19	117	97	120	354
	Permanent disability	0	0	0	0	0	0	0
	Fetal congenital malformation	2	0	0	0	0	0	2
Serious adverse events	Causing or prolonging the patient’s hospital stay	15	78	456	1723	878	1049	4199
	Other clinically significant events	22	102	510	2210	1014	754	4612
Non-serious adverse events		21	157	982	6197	1831	1242	10430
Sum		60	339	1974	10,469	4148	4230	21,225
Number of doses		725,665	2,753,398	3,617,636	33,044,622	15,618,398	12,298,416	68,058,135
Notification rate (cases/100,000 doses)		8.3	12.3	54.6	31.7	26.6	34.4	31.2
Serious adverse event notification rate (cases/100,000 doses)		5.4	6.6	27.4	12.9	14.8	24.3	15.9

**Table 2:** Number of death notifications stratified by age group from 22 March 2021 to 30 September 2023.

Age Range	Number of Doses	Number of Death Notifications	Death Notification Rate (Cases/100,000 Doses)
<5	725,665	2	0.3
5–11	2,753,398	1	0.0
12–17	3,617,636	7	0.2
18–49	33,044,622	222	0.7
50–64	15,618,398	328	2.1
≥65	12,298,416	1065	8.7

**Table 3:** Cumulative reporting of adverse events of special concern in Taiwan from 22 March 2021 to 30 September 2023.

Adverse Events of Special Concern	Number of Reported Cases	Age Range of Cases	Time of Onset
Anaphylaxis	52	10.1~95.5	5 min~2 days
Arrhythmia	64	4.2~94.2	<1~164 days
Acute myocardial infarction	309	25.6~97.9	<1~101 days
Myocarditis/pericarditis	455	3.4~85.8	<1~258 days
Cerebrovascular stroke	746	7.5~96.8	<1~375 days
Facial palsy	237	8.6~88	<1~126 days
Seizure/convulsion	72	7.7~86.5	<1~46 days
Febrile convulsion	5	1~4.1	<1~5 days
Transverse myelitis	4	15.8~61.4	2~23 days
Acute disseminated encephalomyelitis	9	25.8~57	<1~69 days
Guillain–Barré Syndrome, GBS	38	10.2~84.8	1~63 days
Neuromyelitis optica	6	29.6~73.4	3~43 days
Myelitis	6	20.7~81.2	2~46 days
Encephalitis	12	12.6~76.6	1~30 days
Aseptic meningitis	5	25.3~60.9	6~29 days
Optic neuritis	15	13.8~60	<1~35 days
Acute pancreatitis	12	12.1~82.3	<1~41 days
Acute kidney injury	12	25.9~91.6	<1~70 days
Acute liver injury	25	11.6~89.6	<1~70 days
Erythema multiforme	21	2~91.3	<1~77 days
Vasculitis	32	13.7~85.7	<1~100 days
Rhabdomyolysis	20	15.6~83.7	<1~39 days
Arthritis	13	25.4~82.4	2~109 days
Spontaneous abortion	15	26.8~39.5	<1~40 days
Stillbirth	15	22.9~41	3~33 days
Preterm birth	4	29.6~36.3	1~19 days
Idiopathic thrombocytopenic purpura, ITP	80	1.1~100.7	<1~113 days
Thrombosis with thrombocytopenia syndrome, TTS	56	22~95.7	2~44 days
Capillary leak syndrome	1	62.3	3 days
Multisystem inflammatory syndrome in children, MIS-C	2	8.7~12	<1~12 days
Retinal vein occlusion, RVO	26	23.6~88.2	<1~146 days
Retinal artery occlusion, RAO	10	27.9~71	1~57 days
Retinal vascular occlusion	5	56.1~78.7	1~28 days
Deep vein thrombosis	126	13.5~92.8	<1~194 days
Pulmonary embolism	150	17.4~95.5	<1~131 days
Cerebral venous sinus thrombosis	31	27.3~93.6	<1~110 days
Other thrombotic disorder	56	21.6~88.8	<1~243 days

**Table 4:** Cumulative reporting of adverse events of special concern in Taiwan from 26 September 2023 to 31 December 2023.

Adverse Events of Special Concern	Number of Reported Cases	Age Range of Cases	Time of Onset
Acute myocardial infarction	1	18–49	11 days
Myocarditis/pericarditis	1	≥65	18 days
Cerebrovascular stroke	2	50–64	<1~2 days
Facial palsy	1	18–49	<1 days
Guillain–Barré Syndrome, GBS	1	<5	17 days
Acute pancreatitis	1	≥65	15 days
Vasculitis	1	50–64	2 days

Notifications of adverse events of special concern received after vaccination with Moderna XBB.1.5 and Novavax vaccines in Taiwan until 31 December 2023.

**Table 5:** Notification cases of Omicron XBB.1.5 monovalent Moderna XBB.1.5 vaccine (mRNA vaccine) from 26 September 2023 to 31 December 2023.

Moderna XBB.1.5								
Classification of Reported Cases		Number of Reported Cases						Sum
	Age Group (Years)	<5	5–<12	12–<18	18–<50	50–<65	≥65	
	Death	0	0	0	0	0	2	2
	Life-threatening condition	0	0	0	0	0	0	0
Serious adverse events	Permanent disability	0	0	0	0	0	0	0
	Fetal congenital malformation	0	0	0	0	0	0	0
	Causing or prolonging the patient’s hospital stay	1	0	0	1	1	1	4
	Other clinically significant events	0	0	0	0	5	2	7
Non-serious adverse events		1	0	0	1	4	5	11
Sum		2	0	0	2	10	10	24
Number of doses		8813	13,192	7398	151,528	205,779	475,033	861,743
Notification rate (cases/100,000 doses)		22.7	0.0	0.0	1.3	4.9	2.1	2.8
Serious adverse event notification rate (cases/100,000 doses)		11.3	0.0	0.0	0.7	2.9	1.1	1.5

**Table 6:** Notification cases of Novavax vaccine (protein subunit vaccine) from 26 September 2023 to 31 December 2023.

Novavax								
Classification of Reported Cases		Number of Reported Cases						Sum
	Age Group (Years)	<5	5–<12	12–<18	18–<50	50–<65	≥65	
	Die	0	0	0	0	1	3	4
	Life threatening	0	0	0	0	0	0	0
Serious adverse events	Cause permanent disability	0	0	0	0	0	0	0
	Fetal congenital malformation	0	0	0	0	0	0	0
	Causing or prolonging the patient’s hospital stay	0	0	0	8	2	1	11
	Other clinically significant events	0	0	0	6	1	1	8
Non-serious adverse events		0	0	1	9	8	5	23
Sum		0	0	1	23	12	10	46
Number of doses		2	33	10,660	249,608	254,018	128,281	642,602
Notification rate (cases/100,000 doses)		0.0	0.0	9.4	9.2	4.7	7.8	7.2
Serious adverse event notification rate (cases/100,000 doses)		0.0	0.0	0.0	5.6	1.6	3.9	3.6

**Table 7:** Number of death notifications by age group. Timeframes for Moderna XBB.1.5 and Novavax are 26 September 2023 to 31 December 2023 and 8 July 2022 to 31 December 2023, respectively.

	Moderna XBB.1.5			Novavax		
Age Range	Number of Doses	Number of Death Notifications	Death Notification Rate (Case/100,000 Doses)	Number of Doses	Number of Death Notifications	Death Notification Rate (Case/100,000 Doses)
<5	8813	0	0.0	2	0	0.0
5–11	13,192	0	0.0	33	0	0.0
12–17	7398	0	0.0	10,660	0	0.0
18–49	151,528	0	0.0	249,608	0	0.0
50–64	205,779	0	0.0	254,018	1	0.4
≥65	475,033	2	0.6	128,281	3	2.3

**Table 8:** Notification of adverse events after COVID-19 vaccination in Taiwan (deadline for reporting information: 8 February 2023/16:00).

Classification of Reported Cases									
		AstraZeneca	Moderna	MVC	BioNTech	Novavax	Moderna BA.1 Bivalent Vaccine	Moderna BA.4/5 Bivalent Vaccine	Sum
	Accumulated Time Interval	22 March 2021~8 February 2023	8 June 2021~8 February 2023	23 August 2011~8 February 2023	22 September 2021~8 February 2023	8 July 2022~8 February 2023	24 September 2022~8 February 2023	18 November 2022~8 February 2023	
	Death	852	539	64	140	4	13	0	1612
	Suspected severe allergic reaction	25	14	6	3	0	2	0	50
Suspected serious adverse events	Other suspected serious adverse events	3491	2636	340	2541	15	51	53	9127
	Sum	4368	3189	410	2684	19	66	53	10,789
Adverse events		8558	5577	833	5801	40	109	97	21,015
Non-serious adverse events		4190	2388	423	3117	21	43	44	10,226

Data source: Taiwan Centers for Disease Control (https://www.cdc.gov.tw/, accessed on 15 February 2024).

## Data Availability

Data are provided in Supplementary Materials and are available on request.

## Supplementary Materials

The following supporting information can be downloaded at: https://www.mdpi.com/article/10.3390/vaccines12060591/s1, Table S1: Basic dose (including the third dose of BioNTech brand vaccine for children aged 6 months to under 5 years old) monovalent of each brand of COVID-19 vaccine with number of reported cases; Table S2: Monovalent of each brand of basic booster or booster dose and number of COVID-19 vaccine notification cases; Table S3: Number of reported cases of booster dose of bivalent COVID-19 vaccine; Table S4: Notification of adverse events after COVID-19 vaccination in Taiwan.
