# Supplementary material for: COVID-19 Vaccination Reporting and Adverse Event Analysis in Taiwan
Source: Vaccines (Basel). 2024 May 29;12(6):591. doi: 10.3390/vaccines12060591 (PMC11209125; doi:10.3390/vaccines12060591)
Supplement: Supplementary file 1 [file vaccines-12-00591-s001.zip › vaccines-2975263-supplementary.pdf]

Table S1. Definitions of adverse events

Adverse events of special concern	Definition
Anaphylaxis	An acute hypersensitivity reaction due to exposure to a previously encountered antigen. the reaction may include rapidly progressing urticaria, respiratory distress, vascular collapse, systemic shock, and death.
Arrhythmia	Any disturbances of the normal rhythmic beating of the heart or myocardial contraction. Cardiac arrhythmias can be classified by the abnormalities in heart rate, disorders of electrical impulse generation, or impulse conduction.
Acute myocardial infarction	• Myocardial infarction in which the inferior wall of the heart is involved. It is often caused by occlusion of the right coronary artery.<br>• Myocardial infarction in which the anterior wall of the heart is involved. Anterior wall myocardial infarction is often caused by occlusion of the left anterior descending coronary artery. It can be categorized as anteroseptal or anterolateral wall myocardial infarction.
Myocarditis / Pericarditis	• Myocarditis: Inflammatory processes of the muscular walls of the heart (myocardium) which result in injury to the cardiac muscle cells (myocytes, cardiac). Manifestations range from subclinical to sudden death (death, sudden). Myocarditis in association with cardiac dysfunction is classified as inflammatory cardiomyopathy usually caused by infection, autoimmune diseases, or responses to toxic substances. Myocarditis is also a common cause of dilated cardiomyopathy and other cardiomyopathies.<br>• Pericarditis: Inflammation of the pericardium from various origins, such as infection, neoplasm, autoimmune process, injuries, or drug-induced. pericarditis usually leads to pericardial effusion, or constrictive pericarditis.
Cerebrovascular stroke	A group of pathological conditions characterized by sudden, non-convulsive loss of neurological function due to brain ischemia or intracranial hemorrhages. stroke is classified by the type of tissue necrosis, such as the anatomic location, vasculature involved, etiology, age of the affected individual, and hemorrhagic vs. non-hemorrhagic nature.
Facial palsy	Severe or complete loss of facial muscle motor function. This condition may result from central or peripheral lesions. Damage to CNS motor pathways from the cerebral cortex to the facial nuclei in the pons leads to facial weakness that generally spares the forehead muscles. Facial nerve diseases generally results in generalized hemifacial weakness. Neuromuscular junction diseases and muscular diseases may also cause facial paralysis or paresis.
Seizure / Convulsion	Clinical or subclinical disturbances of cortical function due to a sudden, abnormal, excessive, and disorganized discharge of brain cells. Clinical manifestations include abnormal motor, sensory and psychic phenomena. Recurrent seizures are usually referred to as epilepsy or "seizure disorder."
Febrile convulsion	Seizures that occur during a febrile episode. It is a common condition, affecting 2-5% of children aged 3 months to five years. An autosomal dominant pattern of inheritance has been identified in some families. The majority are simple febrile seizures (generally defined as generalized onset, single seizures with a duration of less than 30 minutes). Complex febrile seizures are characterized by focal onset, duration greater than 30 minutes, and/or more than one seizure in a 24 hour period. The likelihood of developing epilepsy (i.e., a nonfebrile seizure disorder) following simple febrile seizures is low. Complex febrile seizures are associated with a moderately increased incidence of epilepsy.
Transverse myelitis	Inflammation of a transverse portion of the spinal cord characterized by acute or subacute segmental demyelination or necrosis. The condition may occur sporadically, follow an infection or vaccination, or present as a paraneoplastic syndrome (see also encephalomyelitis, acute disseminated). Clinical manifestations include motor weakness, sensory loss, and incontinence.
Acute disseminated encephalomyelitis	An acute or subacute inflammatory process of the central nervous system characterized histologically by multiple foci of perivascular demyelination. Symptom onset usually occurs several days after an acute viral infection or immunization, but it may coincide with the onset of infection or rarely no antecedent event can be identified. Clinical manifestations include confusion, somnolence, fever, nuchal rigidity, and involuntary movements. the illness may progress to coma and eventually be fatal.
Guillain–Barre´ Syndrome, GBS	An acute inflammatory autoimmune neuritis caused by T cell- mediated cellular immune response directed towards peripheral myelin. Demyelination occurs in peripheral nerves and nerve roots. The process is often preceded by a viral or bacterial infection, surgery, immunization, lymphoma, or exposure to toxins. Common clinical manifestations include progressive weakness, loss of sensation, and loss of deep tendon reflexes. Weakness of respiratory muscles and autonomic dysfunction may occur.
Neuromyelitis optica	a syndrome characterized by acute optic neuritis; myelitis, transverse; demyelinating and/or necrotizing lesions in the optic nerves and spinal cord; and presence of specific autoantibodies to aquaporin 4.
Myelitis	Inflammation of the spinal cord. Relatively common etiologies include infections; autoimmune diseases; spinal cord; and ischemia (see also spinal cord vascular diseases). Clinical features

	generally include weakness, sensory loss, localized pain, incontinence, and other signs of autonomic dysfunction.
Encephalitis	Inflammation of the brain due to infection, autoimmune processes, toxins, and other conditions. Viral infections (see encephalitis, viral) are a relatively frequent cause of this condition.
Aseptic meningitis	A syndrome characterized by headache, neck stiffness, low grade fever, and CSF lymphocytic pleocytosis in the absence of an acute bacterial pathogen. Viral meningitis is the most frequent cause although mycoplasma infections; rickettsia infections; diagnostic or therapeutic procedures; neoplastic processes; septic perimeningeal foci; and other conditions may result in this syndrome.
Optic neuritis	Inflammation of the optic nerve. Commonly associated conditions include autoimmune disorders such as multiple sclerosis, infections, and granulomatous diseases. Clinical features include retro-orbital pain that is aggravated by eye movement, loss of color vision, and contrast sensitivity that may progress to severe visual loss, an afferent pupillary defect (Marcus-Gunn pupil), and in some instances optic disc hyperemia and swelling. Inflammation may occur in the portion of the nerve within the globe (neuropapillitis or anterior optic neuritis) or the portion behind the globe (retrobulbar neuritis or posterior optic neuritis).
Acute pancreatitis	Inflammation of the pancreas. Pancreatitis is classified as acute unless there are computed tomographic or endoscopic retrograde cholangiopancreatographic findings of chronic pancreatitis (International Symposium on Acute Pancreatitis, Atlanta, 1992). The two most common forms of acute pancreatitis are alcoholic pancreatitis and gallstone pancreatitis.
Acute kidney injury	Abrupt reduction in kidney function. Acute kidney injury encompasses the entire spectrum of the syndrome including acute kidney failure; acute kidney tubular necrosis; and other less severe conditions.
Acute liver injury	A spectrum of clinical liver diseases ranging from mild biochemical abnormalities to acute liver failure, caused by drugs, drug metabolites, herbal and dietary supplements and chemicals from the environment.
Erythema multiforme	A skin and mucous membrane disease characterized by an eruption of macules, papules, nodules, vesicles, and/or bullae with characteristic "bull's-eye" lesions usually occurring on the dorsal aspect of the hands and forearms.
Vasculitis	Inflammation of any one of the blood vessels, including the arteries; veins; and rest of the vasculature system in the body.
Rhabdomyolysis	Necrosis or disintegration of skeletal muscle often followed by myoglobinuria.
Arthritis	Acute or chronic inflammation of joints.
Spontaneous abortion	Expulsion of the product of fertilization before completing the term of gestation and without deliberate interference.
Stillbirth	The event that a fetus is born dead or stillborn.
Preterm birth	Childbirth before 37 weeks of pregnancy (259 days from the first day of the mother's last menstrual period, or 245 days after fertilization).
Idiopathic thrombocytopenic purpura, ITP	Thrombocytopenia occurring in the absence of toxic exposure or a disease associated with decreased platelets. It is mediated by immune mechanisms, in most cases immunoglobulin G autoantibodies which attach to platelets and subsequently undergo destruction by macrophages. The disease is seen in acute (affecting children) and chronic (adult) forms.
Thrombosis with thrombocytopenia syndrome, TTS	Thrombocytopenia occurring in the absence of toxic exposure or a disease associated with decreased platelets. It is mediated by immune mechanisms, in most cases immunoglobulin G autoantibodies which attach to platelets and subsequently undergo destruction by macrophages. The disease is seen in acute (affecting children) and chronic (adult) forms.
Capillary leak syndrome	A condition characterized by recurring episodes of fluid leaking from capillaries into extra-vascular compartments causing hematocrit to rise precipitously. If not treated, generalized vascular leak can lead to generalized edema; shock; cardiovascular collapse; and multiple organ failure.
Multisystem inflammatory syndrome in children, MIS-C	Paediatric multisystem inflammatory syndrome temporally associated with COVID-19
Retinal vein occlusion, RVO	Blockage of the retinal vein. Those at high risk for this condition include patients with hypertension; diabetes mellitus; atherosclerosis; and other cardiovascular diseases.
Retinal artery occlusion, RAO	Sudden ischemia in the retina due to blocked blood flow through the central retinal artery or its branches leading to sudden complete or partial loss of vision, respectively, in the eye.
Retinal vascular occlusion	An eye condition that affects the retina — the light-sensitive layer of tissue in the back of your eye. It happens when a blood clot blocks the main vein where blood flows out of the retina (https://www.nei.nih.gov/learn-about-eye-health/eye-conditions-and-diseases/central-retinal-vein-occlusion-crvo, accessed on 12 May, 2024).
Deep vein thrombosis	The formation or presence of a blood clot (thrombus) within a vein.
Pulmonary embolism	Blocking of the pulmonary artery or one of its branches by an embolus.
Cerebral venous sinus thrombosis	Cerebral venous sinus thrombosis (CVST) occurs when a blood clot forms in the brain's venous sinuses. This prevents blood from draining out of the brain (https://www.hopkinsmedicine.org/health/conditions-and-diseases/cerebral-venous-sinus-thrombosis, accessed on 12 May, 2024).
Other thrombotic disorder	Other disorders in addition to formation and development of a thrombus or blood clot in blood vessels.

Table S2. Basic dose (including the third dose of BioNTech brand vaccine for children aged 6 months to under 5 years old) monovalent of each brand COVID-19 vaccine number of reported cases.

Classification of reported cases		Notification cases of basic dose #					Sum
		AstraZeneca	Moderna	BioNTech	MVC	Novavax	
Serious adverse events	Die	850	411	110	56	2	1429
	Life threatening	126	71	74	12	0	283
	Cause permanent disability	0	0	0	0	0	0
	Fetal congenital malformation	0	1	0	1	0	2
	Causing or prolonging the patient's hospital stay	1354	889	1058	118	2	3421
	Other clinically significant events	1929	860	1154	172	2	4117
Total serious adverse events		4259	2232	2396	359	6	9252
Non-serious adverse events		4307	1580	2785	374	9	9055
Sum		8566	3812	5181	733	15	18307

#Including the third dose of BioNTech brand vaccine for children aged 6 months to under 5 years old.

Table S3. Monovalent of each brand of basic booster or booster dose and number of COVID-19 vaccine notification cases.

Classification of reported cases		Notification cases of basic dose #					Sum
		AstraZeneca	Moderna	BioNTech	MVC	Novavax	
Serious adverse events	Die	1	135	32	8	2	178
	Life threatening	0	47	11	3	0	61
	Cause permanent disability	0	0	0	0	0	0
	Fetal congenital malformation	0	0	0	0	0	0
	Causing or prolonging the patient's hospital stay	2	506	148	23	9	688
	Other clinically significant events	3	289	131	14	6	443
Total serious adverse events (%)		6	977	322	48	17	1370
Non-serious adverse events		9	840	345	56	14	1264
Sum		15	1817	667	104	31	2634

Table S4. Number of reported cases of booster dose of bivalent COVID-19 vaccine.

Classification of reported cases		Notification cases of basic dose #		Sum
		Moderna bivalent BA.1	Moderna bivalent BA.4/5	
Serious adverse events	Die	15	5	20
	Life threatening	2	8	10
	Cause permanent disability	0	0	0
	Fetal congenital malformation	0	0	0
	Causing or prolonging the patient's hospital stay	38	51	89
	Other clinically significant events	13	37	50
Total serious adverse events (%)		68	101	169
Non-serious adverse events		45	63	108
Sum		113	164	277
